# Wearable Electro‐Thermal Haptic Stimulator Driven by a Self‐Powered Tactile Sensor for Realistic Stimulus Replication

**DOI:** 10.1002/advs.76616

**Published:** 2026-07-13

**Authors:** Ey‐In Lee, Chae‐Young Kang, Kyuhyun Hwang, Jongbaeg Kim, Jin‐Woo Park

**Affiliations:** ^1^ Department of Materials Science and Engineering Yonsei University Seoul Republic of Korea; ^2^ School of Mechanical Engineering Yonsei University Seoul Republic of Korea

**Keywords:** electrotactile stimulations, self‐powered tactile sensors, tactile stimulus‐replicating system, thermotactile stimulations

## Abstract

Reproducing realistic tactile sensations is critical for intuitive and immersive human–machine interaction in bio‐mechatronic systems. Here, we present a stimulus‐replicating system that translates real‐world tactile events into biomimetic sensations. The system couples a self‐powered, multimodal tactile sensor—detecting dynamic/static pressure and temperature via hybrid triboelectric and ionic mechanisms—with a wearable stimulator. The stimulator features a co‐located Peltier‐based thermotactile module and a concentric poly(2,3‐diydrothieno‐1,4‐dioxin)‐poly(styrenesulfonate)/polyurethane electrotactile electrode, optimized through Multiphysics simulations for spatially focused receptor activation. This integrated architecture, stabilized by aluminum nitride/polydimethylsiloxane encapsulation and acrylate‐based pressure‐sensitive adhesion, faithfully reproduces sensed mechano‐thermal signatures on the user's skin. Psychophysical tests further show high accuracy in discriminating pressure, temperature, and softness: 81.7% accuracy in discriminating pressure and softness via electrotactile stimulation and 72% accuracy in discriminating temperature via thermotactile stimulation. By moving beyond predefined haptics, our framework establishes a new approach to biomimetic sensation delivery. This technology holds significant promise for applications requiring high‐fidelity tactile replication, including smart prosthetics and tele‐haptics.

## Graphical abstract

We present a stimulus‐replicating system combining a tactile sensor with a wearable Peltier thermotactile module and concentric electrotactile electrode. Signals encoding pressure and temperature drive the stimulators to reproduce mechano‐thermal cues on the skin. AlN/PDMS encapsulation and SA/LA PSA ensure mechanical compliance and thermal efficiency. Psychophysical tests show high accuracy in distinguishing pressure, temperature, and softness. Applications include prosthetics, tele‐haptics, and surgery.

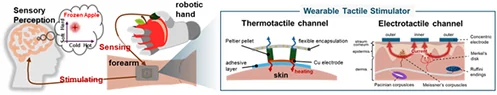

## 1 Introduction

Perception–the brain's real‐time integration of multisensory cues–allows humans to construct an accurate representation of their environment [1, 2]. Through the visual, auditory, tactile, and olfactory senses, humans interpret their surroundings with sufficient sensory input. Since the advent of the Fourth Industrial Revolution, the spread of advanced mechatronic and digital technologies has shifted the focus of mechatronics from simply enhancing device performance to creating user‐centered interfaces that are stable, safe, and intuitive, spawning the field of bio‐mechatronics [3, 4]. As interactions with machines grow more immersive–particularly in augmented/virtual reality (AR/VR), medical prosthetics, and tele‐robotics–high‐fidelity tactile communication has become essential. Because sight and sound cannot fully convey key physical properties, touch must be further developed to provide direct information on object compliance, surface topology, slip, and temperature [5, 6, 7, 8].

Meeting this need for tactile communication requires a stimulus‐replicating system that integrates tactile sensors with stimulators capable of reproducing the detected stimuli on the user's skin in real time [9, 10, 11]. In 2023, surgery‐assisted robotics with a tactile stimulus‐replicating system succeeded in Japan for the first time in the world [12, 13]. Such a system reduced average forces and completion time with enhanced surgical accuracy and success rates compared to operations without a stimulus‐conveying system [14]. Also, current commercial prosthetic hands are generally driven by electromyography (EMG) signals that encode a user's intent, while discrete pressure sensors embedded at the fingertips detect external loads [15, 16]. The sensor data either modulates the grasping force automatically or activates a stimulator to provide mechanical cues to the residual limb. The Ability Hand (Psyonic, Inc.), for instance, houses small vibrators that press against the stump and vary the vibration amplitude according to the fingertip pressure [17].

However, current system primarily focuses on stimulus detection rather than sensory emulation; by encoding only rudimentary features like onset and intensity, they fail to bridge the gap between symbolic tactile feedback and a truly natural haptic experience [18, 19]. When the feedback is derived from biomimetic, skin‐like sensors and delivered according to the skin's own transduction principles, it preserves the contextual cues and enables the delivery of realistic sensations. Such capabilities move haptics beyond the predefined sensations typical of conventional technologies, permitting safe interaction with real, unpredictable objects and unlocking new opportunities in surgical training, industrial tele‐maintenance, and neurorehabilitation—where errors can carry serious consequences.

Authentic haptic delivery necessitates a multimodal approach, as temperature provides a sensory cue as critical as pressure for reconstructing a natural sense of touch. In 2023, researchers introduced a system that mapped a phantom hand onto the residual limb, detected external thermal events with dedicated sensors, and replicated temperature changes in real time using a commercial Peltier element for the first time [20]. Moreover, J.‐H. Kim et al. (2024) developed a skin‐integrated thermo‐haptic stimulating system by incorporating commercial resistive temperature sensors into the PSYONIC Ability Hand [17]. Collectively, these studies underscore the need for multimodal—pressure and thermal—sensing and stimulating in advanced prosthetics and tele‐robotic grippers [21, 22]. Nonetheless, to realize a truly advanced stimulus‐replicating system, rather than merely integrating commercial separate sensors, functionally integrated, single‐unit multimodal sensors should be employed with minimized energy consumption and bulkiness of the system.

Regarding pressure‐based stimulation, the two primary mechanisms can be considered: mechanotactile and electrotactile [23, 24]. Mechanotactile methods mechanically deform the skin through wearable actuators [25]. However, replicating diverse tactile cues is challenging because they focus primarily on simple normal forces or vibrations. Achieving realistic feedback instead requires artificial stimuli that emulate the encoding strategies of cutaneous receptors. These receptors transduce external inputs into electrical signals that propagate to the nervous system [26]. The electrotactile stimulation–delivering controlled electrical currents through wearable electrodes–therefore offers a more biomimetic route [27]. Merkel discs and Meissner corpuscles, located at the dermal‐epidermal junction, specialize in static and dynamic touch, respectively. This region is relatively superficial compared to the deeper dermal layers where the other two mechanoreceptors, Pacinian corpuscles and Ruffini endings, are situated [28]. Applying an electric field to the skin directly stimulates the afferent nerve fibers within the dermis [29]. The resulting current flow through the tissue depolarizes nearby nerve endings, eliciting action potentials that the brain interprets as tactile sensations. By modulating electrical parameters—including frequency, intensity, and pulse width‐ this method can induce a diverse spectrum of sensations, such as vibration, pressure, tingling, and texture, while simultaneously influencing comfort levels and the precision of intensity discrimination [23, 30, 31].

In this study, we demonstrate the stimulus‐replicating system that integrates a self‐powered tactile sensor with a wearable tactile stimulator. The sensor, previously developed by our group, simultaneously detects dynamic and static pressure and temperature within a single unit [32]. These signals are then used to drive the wearable stimulator, which delivers corresponding mechanical and thermal cues to the user. The stimulator consists of two key components: (i) a conformable thermotactile module, comprising a Peltier element with a flexible encapsulating layer for both heating and cooling, and (ii) an electrotactile electrode fabricated from PEDOT:PSS. The electrode's geometry was optimized through Multiphysics simulations to isolate the activation of a specific receptor class, purposefully suppressing crosstalk to neighboring tactile receptors for a more distinct haptic sensation. An acrylate‐based pressure‐sensitive adhesive secures both elements, ensuring stable and consistent skin contact. By detecting external stimuli and faithfully reproducing them on the user's skin, this integrated system validates the proposed stimulus‐replicating concept. Psychophysical tests confirmed its effectiveness, showing that the electrotactile stimulator conveyed pressure and softness cues with a mean accuracy of 81.7%, while the thermotactile stimulator conveyed thermal cues with 72% accuracy.

## 2 Results and Discussion

### 2.1 Concept and Functions of the Stimulus‐Replicating System

This study implements a stimulus‐replicating system by coupling a self‐powered tactile sensor with a wearable tactile stimulator as shown in Figure 1. When the sensor encounters an external stimulus–for example, during the grasp of an apple–it simultaneously records dynamic and static pressure as well as temperature (① in Figure 1). The resulting multimodal signals are processed to drive the wearable stimulator (② in Figure 1), which then recreates the sensed mechanical and thermal cues on the user's skin (③ in Figure 1). Based on the information captured by the sensor, the stimulation evokes sensory perception in the user, thereby conveying information about the external stimulus (④ in Figure 1).

**FIGURE 1:**
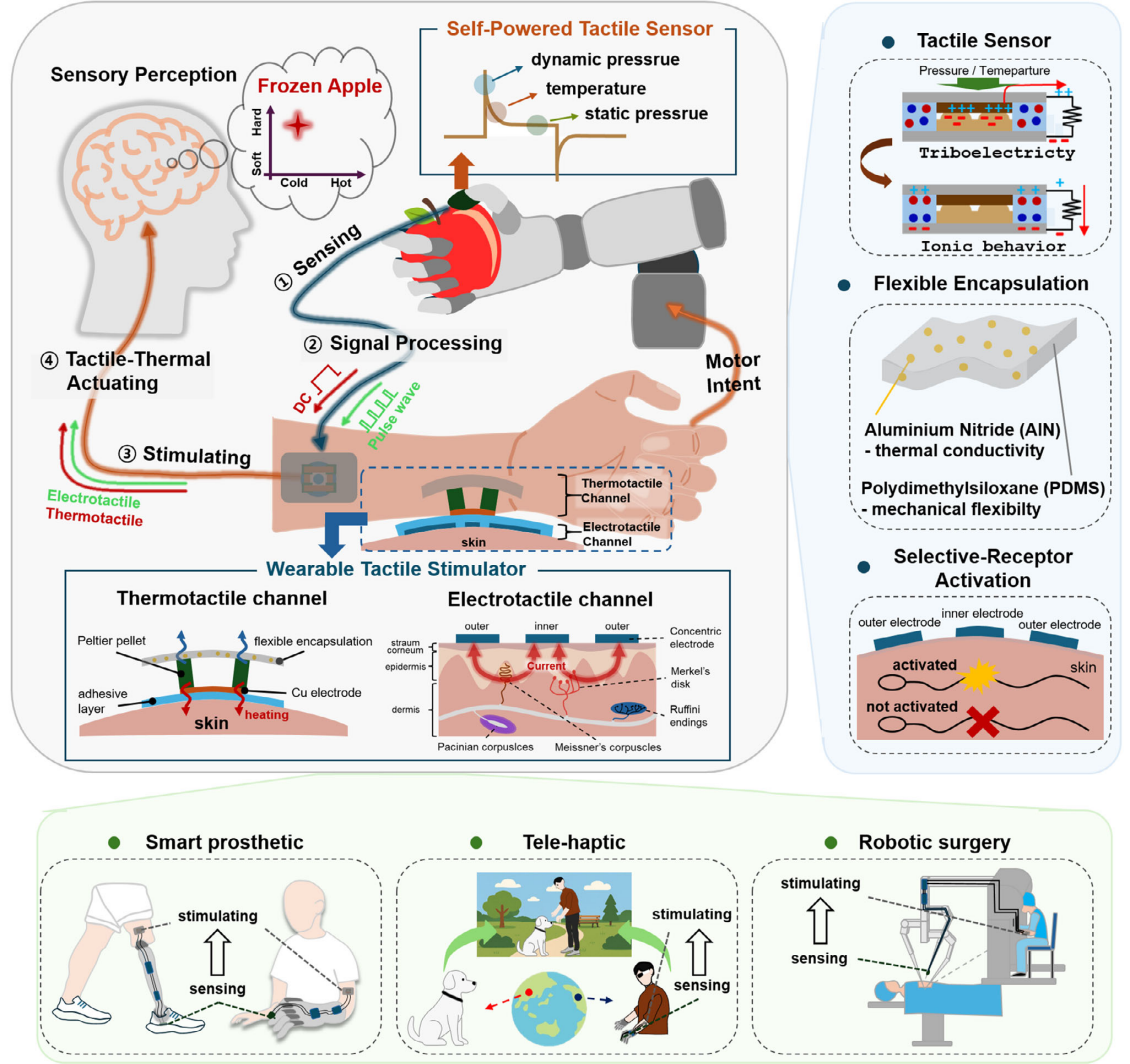
Conceptual illustration of the stimulus‐replicating system. Schematic illustration of the stimulus‐replicating system, which consists of a self‐powered tactile sensor and a wearable tactile stimulator.

The tactile sensor, previously reported by our group, combines triboelectric charge generation with ionic mechanisms, harvesting its own operating power while simultaneously sensing pressure and temperature [32]. The multimodal sensor's self‐powering capability enables a compact, energy‐efficient system. The wearable stimulator incorporates two co‐located actuation channels. First, a Peltier element bonded to a compliant AlN/PDMS composite encapsulating layer provides bidirectional heating and cooling under a DC bias while maintaining intimate contact with curved skin surfaces via an acrylate pressure‐sensitive adhesive. In parallel, a concentric PEDOT:PSS/PU electrode–whose geometry is optimized to maximize the stimulation of superficial mechanoreceptors while minimizing the unintended activation of deeper receptors– delivers pulsed voltage that reproduces mechanical transients that the thermal pathway cannot convey.

Because the sensor and the stimulator share the same stimulus, the detected mechanical‐thermal signals are thoroughly reproduced on the skin, yielding tactile‐thermal actuation that the user perceives as realistic combinations of two modalities. This integrated architecture is well suited to emerging bio‐mechatronic applications. In smart prosthetic hands, it can relay fingertip pressure and temperature to the residual limb, restoring delicate feedback and improving grip stability. In a tele‐haptic system, it transmits remote contact events to an operator's glove, enhancing situational awareness in hazardous environments and allowing users at distant locations to share tactile information. Finally, in robotic surgery, it augments the surgeon's visual feed with compliance and temperature cues, potentially reducing iatrogenic injury and improving procedural precision.

### 2.2 Design of the Electrotactile Electrode

An electrotactile stimulation is a non‐invasive method for delivering tactile sensations by applying microcurrents to the skin [27, 33]. When the skin is exposed to mechanical stimuli above the threshold, cutaneous mechanoreceptors respond to specific inputs and generate action potentials by opening ion channels that allow ion movement. The diffusion of these ions into adjacent regions enables signal conduction along nerve fibers. Because the transmission of tactile signals to the central nervous system is fundamentally electrical, applying microcurrent directly to the skin offers a straightforward and effective method for generating artificial tactile sensations. Mechanoreceptors that are naturally responsive to mechanical stimuli can also be activated by electrical stimulation. To enable such stimulation, wearable electrodes are required, and to ensure stable stimulation with minimal power consumption, low‐impedance electrodes are preferred [34].

A polymer‐based electrode of PEDOT:PSS, providing electrical conductivity, and PU, providing mechanical compliance, was selected for its low contact resistance with the skin and robust mechanical adhesion [35]. Based on the patternability of polymer electrodes, a concentric design was adopted, which has been shown to offer a more comfortable and realistic sensation while reducing the likelihood of undesirable sensations such as pain, pinching, or pricking [36, 37]. When electrical stimulation is applied to the skin, the resulting current penetrates the underlying layers where various mechanoreceptors are located. The four major mechanoreceptors–Merkel discs, Meissner corpuscles, Ruffini endings, and Pacinian corpuscles–are distributed at different depths and respond to different types of mechanical input. Among these, Merkel discs and Meissner corpuscles, which are responsible for detecting static and dynamic pressure, respectively, are located near the dermal‐epidermal interface [26]. These superficial receptors are the primary targets for spatially focused activation, necessitating optimization of the electrode geometry.

The activating function, defined as the second spatial derivative of the electric potential along the longitudinal axis of a nerve fiber, is used to estimate the excitability of neural tissue [38]. In the context of axonal stimulation, regions with a positive activating function are likely to generate an action potential, with higher values corresponding to a greater probability of nerve activation and increased sensitivity to electrical stimulation [39]. As illustrated in Figure 2a, when current is applied through the concentric electrode, the measured voltage distribution is focused on the electrode center. From this voltage profile, the activating function is computed along the depth direction of the skin. Regions with a positive activating function, shown in red, indicate depolarization and potential neural activation, whereas regions with a negative value, shown in green, indicate hyperpolarization and reduced excitability.

**FIGURE 2:**
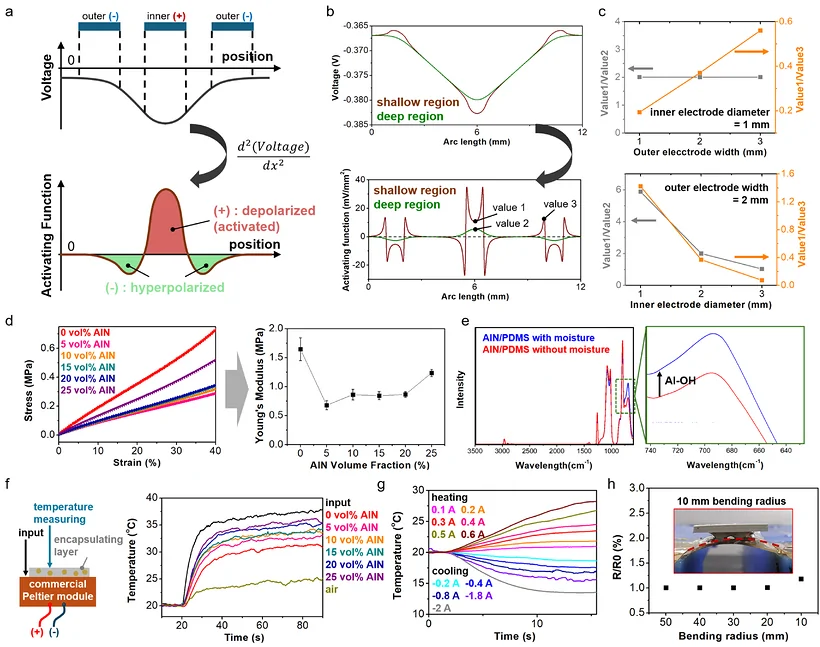
Optimization and characterization of the electrotactile and thermotactile stimulator. (a) Schematic of voltage distribution and activating function. (b) Measured voltage distribution and activating function with concentric electrode. (c) Parametric analysis of electrode size on selectivity. (d) Stress‐strain curves of AlN/PDMS composites with varying AlN contents (N = 4). Error bars represent the standard deviation, reflecting the combination of instrumental uncertainty at low‐load regimes and statistical uncertainty arising from minor variations in composite homogeneity. (e) FTIR spectra of AlN/PDMS composites fabricated under different moisture conditions. (f) Thermal conductivity of AlN/PDMS composites with different AlN contents. (g) Heating and cooling performance of the thermotactile stimulator. (h) Resistance variation under different bending radii.

Using Comsol Multiphysics simulations, the concentric electrode geometry was optimized to target mechanoreceptors located in the superficial layers of the skin (Figure S1). The skin was modeled as a three‐layer structure comprising the stratum corneum, the epidermis, and the dermis. On the top of the skin, a concentric electrode configuration with an inner electrode and an outer electrode was placed, where current flowed into the inner electrode and exited through the outer electrode. Since the targeted mechanoreceptors are located near the surface, the simulations evaluated both the voltage distribution and the activating function values in shallow and deep layers (Figure 2b). To ensure a meaningful comparison, the activating function was extracted from the region directly beneath the center of the inner electrode while excluding sharp peaks at the electrode edges. Three representative values were used: (Value 1–the activating function in the shallow region just below the center of the inner electrode. Value 2–the activating function in the deep region just below the center of the inner electrode. Value 3–the activating function in the shallow region just below the outer electrode).

A higher Value 1 compared to Value 2 indicates greater spatial selectivity for the targeted mechanoreceptors, whereas a higher Value 1 compared to Value 3 reflects stronger spatial focusing of the stimulus within the target area. To further analyze these characteristics, simulations were performed by systematically varying the sizes of the inner and outer electrodes, and the corresponding activating function profiles are presented in Figure S2. As shown in Figure 2c, increasing the outer electrode width enhances lateral focusing but does not significantly alter selectivity, whereas reducing the inner electrode diameter improves both selectivity and focality. In conclusion, the concentric design with a smaller inner electrode and a wider outer electrode enables more precise, receptor‐specific stimulation.

Considering the two‐point discrimination threshold of the human forearm (∼4 cm) [40], where the electrotactile electrode is intended to be applied, the total electrode diameter was fixed at 2 cm. An inner electrode diameter of 2 mm and a corresponding outer electrode width of 8 mm were selected. The mold used to fabricate the outer electrode is shown in Figure S3a. The activating function of the design is presented in Figure S3b, along with performance metrics (Value1 1/Value 2: 1.947, Value 1/Value 3: 0.6), demonstrating the electrode's effectiveness in delivering spatially focused electrotactile stimulation.

### 2.3 Design and Performance of the Thermotactile Stimulator

A thermotactile stimulation enables users to perceive the thermal characteristics of objects, thereby enriching the realism of tactile feedback beyond pressure alone. Among the two representative thermotactile stimulation methods—Joule heater‐based and Peltier element‐based stimulation—only the latter provides both heating and cooling owing to its bidirectional heat transfer mechanism [19]. When an electric current flows through a Peltier device, charge carriers in both p‐ and n‐type semiconductors migrate toward the junction, transferring heat from the heat sink to the junction, which results in surface heating. Reversing the current direction causes heat to flow away from the surface, enabling cooling. For this modality‐matched stimulator to effectively deliver thermal cues to the skin, it must exhibit both mechanical and thermal compliance, which requires a flexible encapsulated Peltier element.

The encapsulating layer plays a critical role in mechanical flexibility and thermal conductivity. Typical elastomeric encapsulants have extremely low thermal conductivity, impeding heat transfer and diminishing thermal performance. Most flexible Peltier devices use such soft polymers, limiting the efficiency of heat exchange between the device and the external environment [41, 42]. To enhance both mechanical and thermal performance, AlN, known for its high thermal conductivity, was incorporated into the silicon‐based elastomer, PDMS. Figure 2d presents the mechanical properties of AlN/PDMS composites with various AlN volume fractions (0–25 vol%), with data derived from four samples per condition (N = 4). The error bars presented in the right figure originate from minor inhomogeneities introduced during the manufacturing process of the composites, as well as difficulty in measuring extremely low loads. Compared with neat PDMS (0 vol% AlN), adding AlN reduces the stress required for the same strain, lowering the elastic modulus. Specifically, the modulus of pristine PDMS (1.7 MPa) decreases to approximately 0.8 MPa at 5–20 vol% AlN. However, at 25 vol%, the modulus rises again to 1.19 MPa. Contrary to expectations that rigid AlN fillers would enhance the mechanical stiffness, a decrease in modulus was observed. This phenomenon necessitates further analysis of the filler‐matrix interactions.

Since the AlN/PDMS composites were fabricated under ambient air (40% RH), we investigated the possible influence of moisture on the reduction of Young's modulus by comparing composites prepared in ambient air with those prepared in a glove box with almost 0% RH. Figure S4a presents the mechanical properties of AlN/PDMS composites prepared under different fabrication conditions. Unlike the moisture‐exposed samples, the composites prepared in the glove box (without moisture exposure) exhibited no reduction in Young's modulus, indicating that exposure to moisture plays a critical role in decreasing the modulus.

Fourier transform infrared (FTIR) spectra of the AlN/PDMS composites prepared in ambient air (40% RH) and in a glove box (without moisture) are shown in Figure 2e. To determine the peak position associated with AlN, we further compared the FTIR spectra of neat PDMS and of composites with varying AlN contents (Figure S4b), confirming that the characteristic peak observed around 700 cm^−^^1^ originates from the presence of AlN. By magnifying the spectral region near 700 cm^−^^1^, the band is resolved into two components: a strong, sharp peak centered at 690 cm^−^^1^ may be assigned to the vibration of the Al─N bond [43] and a weak, broad peak centered at 726 cm^−^^1^ may be assigned to the bending vibration of the Al─OH and Al─O bond (Figure 2e) [44]. Comparing the peak intensity ratio at 690 and 726 cm^−^^1^ for composites prepared in ambient air vs. the glove box shows a much stronger 726 cm^−^^1^ component in the moisture‐exposed sample (Figure S4c). This indicates that hydrolysis of AlN occurs under ambient air processing, forming Al─OH groups on the AlN surface. The formation of Al─OH groups can interfere with PDMS curing via a cure‐retarding reaction. Surface ‐OH groups on aluminum hydroxide react with the Si─H groups of the hydride cross‐linker to form Si─O─Al bonds, thereby reducing the number of effective cross‐links [45]. This reduction in cross‐linking density decreases the Young's modulus of the AlN/PDMS composite. When the AlN loading exceeds ≈ 25 vol%, the classical particle‐reinforcement effect dominates, and the modulus rises despite the cure‐retarding reactions [46]. Beyond this threshold, filler stiffening overtakes the modulus‐reducing effect of diminished cross‐link density.

The heat‐transfer performance also improves with increasing AlN content. To quantify this, the temperature on the outer surface of the encapsulating layer placed above a commercial Peltier module was measured. For all samples, the initial temperature was set to 20°C, and the Peltier module was operated to raise its surface temperature to 37°C over 70 s. The encapsulating layer (1 mm thick) was placed above the Peltier, and the temperature was monitored on its top surface. As expected, higher AlN content resulted in more efficient heat transfer as shown in Figure 2f. Neat PDMS showed a surface temperature change of approximately 10°C, while the 5 vol% composite reached 12°C, the 10 and 15 vol% composites reached 13.5°C, and both 20 and 25 vol% composites achieved 15.5°C in response to a 17°C input. These values significantly exceeded that of a 1 mm air gap, which exhibited only a 5°C change.

To quantitatively evaluate the thermal responsiveness of the encapsulating layer, the time constant (τ)‐defined as the time required to reach 63.2% of the steady‐state temperature–was analyzed for various AlN loadings from Figure 2f. The τ values were measured as 10.0498, 8.1913, 7.48, 7.189, 7.133, and 7.26 s for AlN contents of 0, 5, 10, 15, 20, and 25 vol%, respectively. While these values exceed the response time of the bare Peltier module surface (4.53 s), the addition of 20 vol% AlN effectively reduced the thermal lag by approximately 29% compared to neat PDMS (10.05 s). Notably, the thermal response plateaued beyond 15 vol%, confirming that 20 vol% AlN provides an optimal balance between rapid thermal feedback and mechanical flexibility.

In conclusion, incorporating AlN into PDMS improves both the mechanical flexibility and the thermal conductance of the encapsulating layer. Although a sudden increase in the elastic modulus occurs at 25 vol% AlN, the thermal performance plateaus between 20 and 25 vol%. Therefore, considering the trade‐off between flexibility and heat conduction, the composite with 20 vol% AlN is optimal for use as the encapsulating layer of the thermotactile stimulator.

Figure 2g shows the heating and cooling performance of the thermotactile stimulator. By applying current of varying amplitude and polarity, the degree of thermal stimulation can be precisely controlled. Higher current amplitudes result in greater temperature changes from a 20°C baseline, while reversing the current polarity switches the mode from heating to cooling. To ensure suitability for wearable applications, the flexibility of the thermotactile stimulator was evaluated. Figure 2h presents resistance variations under different bending radii. The inset image shows the stimulator in the flat and bent states with a bending radius of 10 mm. Using the flat‐state resistance as a reference, we measured resistance under various bending conditions. When bent to a 20 mm radius, the resistance remained nearly unchanged, indicating mechanical and electrical stability; at 10 mm, the resistance increased by approximately 18%.

Because the stimulator is intended to be mounted on the forearm, where the average bending radius is approximately 20–30 mm [47], its durability under repetitive mechanical deformation was further tested. Figure S5 shows the results of cyclic bending tests at a 20 mm radius. The resistance increased during bending and returned to its baseline value in the flat state. After 120 bending cycles, the resistance remained stable, confirming the mechanical robustness and reliability of the thermotactile stimulator for wearable use.

Moreover, to evaluate the long‐term thermal reliability of the AlN/PDMS encapsulating layer, a thermal cycling test consisting of 100 cycles between 25°C and 55°C was conducted. To visually verify the effects of this thermal cycling, we examined the cross‐sectional morphology of the layer using scanning electron microscopy (SEM) (Figure S6a). The absence of any interfacial gaps, voids, or delaminations between the AlN fillers and the PDMS matrix confirms the layer's exceptional resistance to thermal fatigue, which typically arises from the mismatch in the coefficients of thermal expansion (CTE) between the two materials. The distinct distribution of the AlN fillers and the PDMS matrix was further verified using cross‐sectional SEM‐EDS mapping.

To ensure that the macroscopic thermal properties of the encapsulating layer were preserved, we compared the transient temperature profiles of the thermotactile module under a thermal input ranging from 20°C to 37°C, both before and after the thermal cycling test. As shown in Figure S6b, the heating curves are nearly identical, demonstrating that the structural stability of the layer translates into consistent and robust thermal transfer capabilities under repetitive thermal stress.

### 2.4 Integrated Wearable Stimulators

An adhesive layer was introduced between the electrotactile electrode and the thermotactile stimulator to ensure stable integration of the two components while maintaining skin‐attachable properties. The adhesive is an acrylate‐based pressure‐sensitive adhesive (PSA) composed of SA, LA, and urethane diacrylate (UDA). UDA is a long‐chain oligomer that forms a bottlebrush polymer backbone, while SA and LA are crosslinked to the UDA chain [48]. The adhesive properties of the layer–defined by the balance of tackiness, peel adhesion, and cohesion–were tuned by adjusting the ratio of SA (long alkyl side chain) to LA (short alkyl side chain). The crystalline melting transition of the layer varies with composition; above the melting temperature, the material exhibits higher flowability, whereas below the transition temperature it remains in a rigid phase [49]. To achieve sufficient adhesion under ambient conditions, SA and LA were combined to yield a melting temperature below room temperature. Differential scanning calorimetry (DSC) results (Figure 3a) confirmed that the SA/LA mixtures exhibited melting transitions below 20°C. With an SA fraction of 70 wt.%, the adhesive layer exhibited a solid phase prior to UV curing (Figure S7a), while formulations with lower SA content formed adhesive layers after UV curing.

**FIGURE 3:**
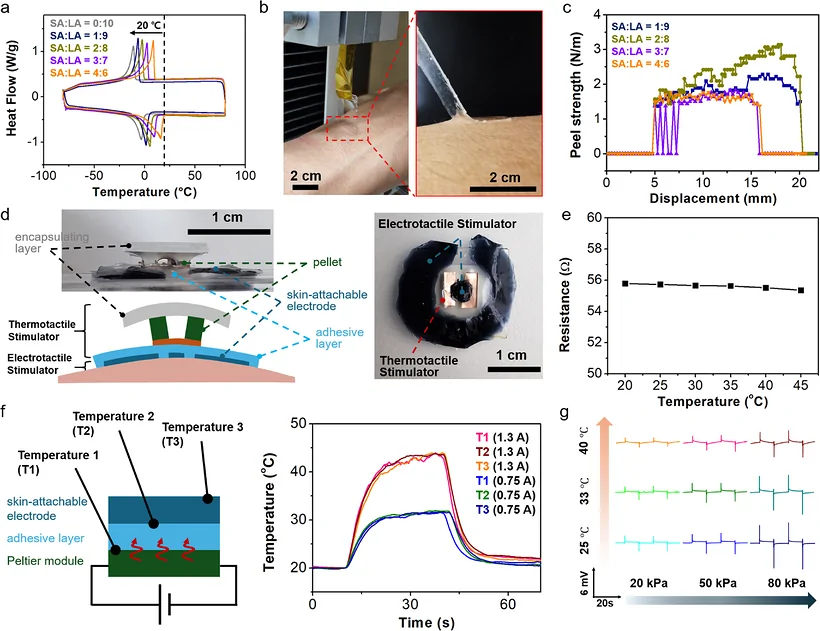
System integration and performance validation of the sensor and stimulator. (a) DSC data of SA/LA adhesive mixtures. (b) Photograph of the SA/LA adhesive layer being peeled off from the skin. (c) Adhesion properties of SA/LA adhesives. (d) Overall composition of the integrated wearable stimulator. (e) Resistance variation of the skin‐attachable electrode under temperature changes. (f) Temperature comparison across different layers. (g) Tactile sensing performance of the sensor under various pressure and temperature conditions.

The adhesive properties were further evaluated using a 90‐degree peel test. As shown in Figure 3b, the adhesive layer was attached to the forearm by gentle pressing and then peeled off at a controlled speed of 10 mm min^−1^. The adhesive layers, each 1.5 cm in width, exhibited peel strengths of approximately 1.5–2 N m^−1^ (Figure 3c). A layer thickness of 300 µm was found to be optimal; as shown in Figure S7b, peel tests could not be performed reliably with thinner layers (200 µm) due to insufficient mechanical integrity. Under tensile loading, layers with SA:LA ratios of 2:8 and 4:6 exhibited increasing load‐bearing capacity, whereas the 3:7 composition failed to sustain any load, indicating weak and unstable cohesion. At a thickness of 300 µm, the layers displayed a stable peel‐strength profile with a clear plateau region. Based on these results, the SA:LA ratio of 2:8 was selected, as it exhibited the highest peel strength among the tested compositions.

The electrotactile stimulator must be in direct contact with the skin to apply current effectively. To deliver both thermal and electrical sensations to the same location, the thermotactile stimulator was vertically stacked on top of the electrotactile stimulator. As illustrated in Figure 3d, the adhesive layer serves a dual purpose: it not only bonds the two components but also serves as a shared substrate. The thermotactile stimulator—comprising a Peltier element and a flexible encapsulating layer—is positioned above the adhesive layer, while the skin‐attachable electrode is positioned below it, forming the direct skin interface.

To evaluate the stability of the electrotactile electrode under thermal stimuli generated by the thermotactile stimulator, the electrode resistance was measured at temperatures between 20°C and 45°C (Figure 3e). The electrode maintained a constant resistance of approximately 55.8 Ω across the tested range, indicating stable electrical properties. Moreover, to verify that the thermal stimuli form the thermotactile stimulator effectively reach the skin without significant heat loss through the adhesive layer and the electrode layers, we measured the temperatures at three locations using a commercial temperature sensor: directly above the Peltier module (T1, input stimulus temperature), between the adhesive layer and electrode (T2), and above the skin‐attachable electrode (T3, perceived skin temperature). As shown in Figure 3f, when currents of 0.75 and 1.3 A were applied to the Peltier module, the temperatures T1, T2, and T3 converged to nearly identical values, confirming efficient thermal transfer to the skin. These results, combined with the data in Figure 3e, collectively demonstrate that the two stimulators do not interfere with each other's performance and that the integrated system maintains stable, independent functionality for both modalities. Furthermore, the established biocompatibility of the skin‐attachable electrode and adhesive layer ensures the device is safe for direct skin contact [35, 49].

Figure 3g shows the simultaneous sensing performance of the tactile sensor under combined dynamic pressure, static pressure, and temperature stimuli. We tested three discrete pressure levels (20, 50, and 80 kPa) at three different temperatures (25°C, 33°C, and 40°C), with 33°C selected to represent average human skin temperature. The sensor output exhibited distinct features for each modality: a peak‐like voltage corresponded to dynamic pressure, a sustained voltage plateau indicated the static pressure, and a decaying voltage profile represented temperature. As applied pressure increased, the amplitudes of both the peak and sustained voltages increased accordingly. Conversely, as temperature increased, the decay time of the thermal signal lengthened while its overall voltage magnitude decreased. These results are consistent with the previously reported hybrid sensing mechanism, which combines triboelectric charge generation and ionic conduction, thereby confirming its multimodal operation [32]. Furthermore, we successfully decoupled these signals using an analytical model of the voltage profile, which verifies the sensor's capability for simultaneous dynamic/static pressure and temperature detection.

### 2.5 Electrotactile Stimulation

The optimized electrotactile electrode design was attached to the forearm with the aid of ethanol to ensure good skin contact, as shown in Figure 4a. Electrochemical impedance spectroscopy (EIS) was performed to confirm stable electrode‐skin contact. As shown in Figure 4b, the system can be represented by an equivalent circuit consisting of one capacitor and two resistors. The phase response exhibits a minimum of approximately −80° near 1 kHz and approaches 0° at low (0.1 Hz) and high (1 MHz) frequencies. The dominance of the resistive component at low and high frequencies indicates that the circuit can be modeled as a series resistor in combination with a parallel resistor‐capacitor network (Figure 4c). The parallel RC element represents the electrode‐skin interface, incorporating contact resistance and ionic charge transfer [50]. Consistent results were obtained across multiple subjects (Figure S8), confirming the same equivalent electrical circuit behavior. Although the skin's electrical characteristics are more complex in reality, the series resistor can be interpreted as representing the inner skin layer, where ions can move more freely [51].

**FIGURE 4:**
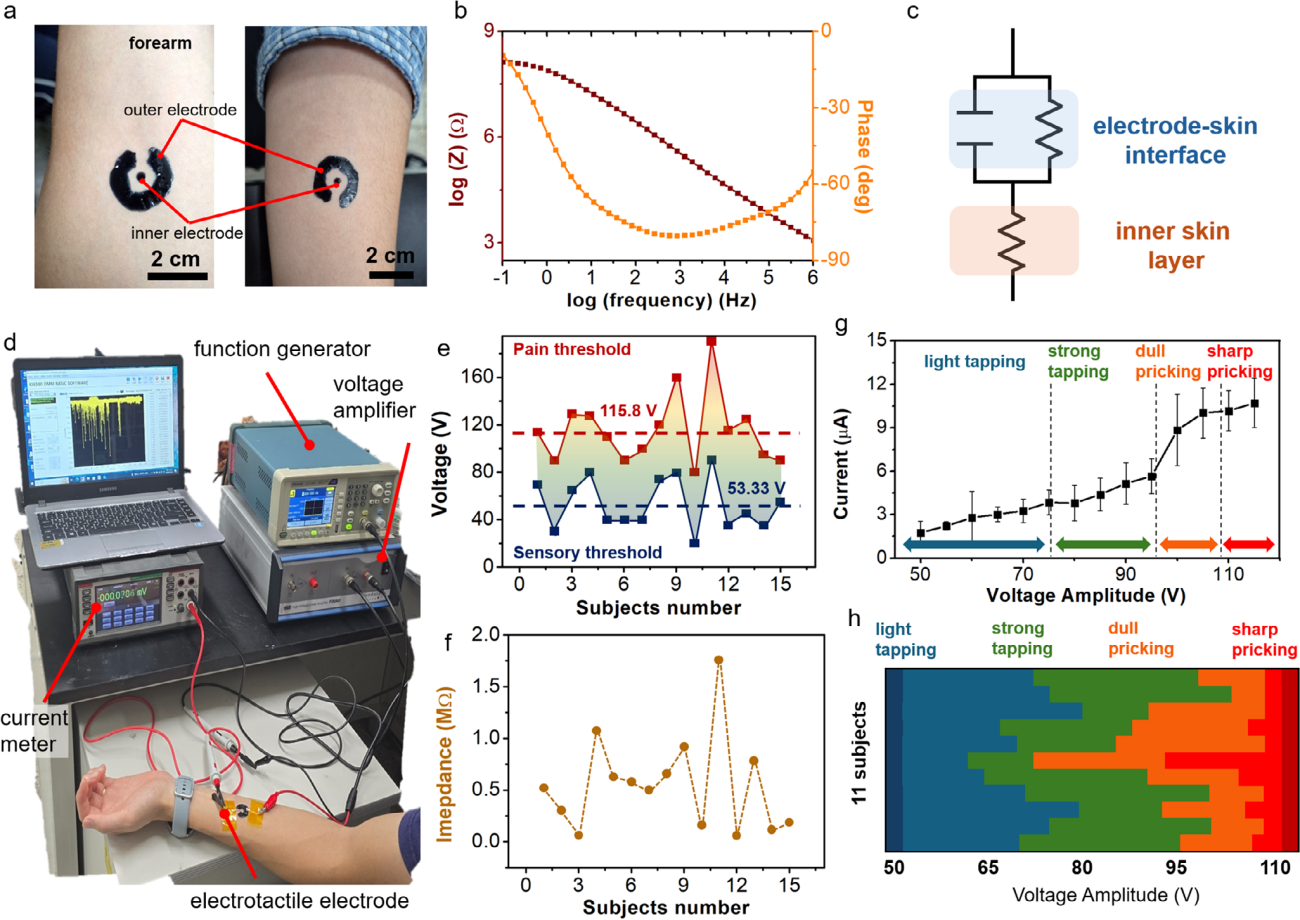
Electrical and psychophysical characterization of the electrotactile stimulator. (a) Photograph of the electrotactile stimulator conformably attached to the forearm. (b,c) EIS measurement and equivalent circuit model of the electrode‐skin interface. (d) Experimental setup for the electrotactile stimulation with a voltage amplifier. (e) Sensory and pain thresholds measured across subjects. (f) Correlation between skin impedance and voltage thresholds. (g) Current response under different applied voltage amplitudes (N = 15). Error bars represent the standard deviation, reflecting statistical uncertainty due to inter‐subject variability in skin impedance and subjective tactile perception. (h) Subjective tactile sensations corresponding to various voltage amplitudes.

For stimulation, a pulse‐wave voltage generated by a function generator was amplified 20 fold using a voltage amplifier and then applied to the electrode attached to the forearm (Figure 4d). Pulse‐wave voltages with a fixed frequency of 1 Hz were generated with tunable amplitude and pulse width (Figure S9a). A pulse width of 1 ms was used to determine sensory and pain thresholds at various amplitudes. Each 1 Hz stimulus was delivered three times for 3 s at a given amplitude. The average minimum voltage amplitude at which eight subjects (9 male, 6 female, age 21–35) began to perceive a tactile sensation was 53.33 V, while the average maximum amplitude at which discomfort was reported was 115.8 V (Figure 4e). Threshold values for individual subjects are summarized in Figure S10. Because tactile sensation is inherently subjective, both sensory and pain thresholds varied among subjects. As demonstrated in Figure 4e and Figure S10, the required voltage amplitude exhibits significant inter‐subject variability. However, both thresholds showed a strong correlation with the skin impedance measured at 1 kHz (Figure 4f). When considering the impedance components of the entire system, the user's skin impedance, which is influenced by individual skin conditions, acts as the most dominant factor in determining these voltage thresholds. Therefore, for practical deployment, calibrating and modulating the applied voltage based on the user's skin impedance can ensure consistent and safe tactile sensations across different individuals.

The resulting current was recorded to quantitatively analyze the relationship between applied voltage and sensory perception. A 1 ms pulse width was applied (Figure S9b), and the corresponding peak‐like current response was modeled using an equivalent circuit consisting of two resistors (20 MΩ, 1 kΩ) and one capacitor (1 nF), derived from the subjects’ EIS data (Figure S9c,d). The measured current increased proportionally with voltage amplitude and exhibited a peak‐like profile consistent with the circuit model (Figure S11). Sensory and pain thresholds averaged 53.55 and 115.8 V, respectively, and subject‐specific values were normalized to a common scale for tactile sensation analysis. As shown in Figure 4g, the current amplitude increased linearly with the subjective tactile sensations reported (N = 15). The error bars arise from inter‐subject variability in skin impedance, as well as differences in subjective tactile perception when identical normalized voltages are applied.

Despite the application of a high voltage reaching the pain threshold (average 115.8 V), the system ensures high electrical safety for human subjects. The measured peak current at this threshold remains below 12 µA, resulting in a maximum current density of 318.46 µA cm^−2^ at the inner electrode interface. These values are significantly lower than the safety limits [52]. This confirms that the high voltage, low current approach effectively evokes the desired tactile sensations while maintaining a substantial safety margin for wearable applications.

Tactile sensations were further assessed under a broad spectrum of voltage intensities. Between 50 and 75 V, subjects described the sensations as light tapping on the forearm. Above 75 V, the sensation intensified and transitioned to strong tapping, and at 95 V the perception shifted from tapping to dull pricking. With further increases, sharp pricking was reported, marking the onset of pain. Voltage amplitudes corresponding to each type of tactile sensation were determined based on subjective reports from eleven representative subjects (Figure 4h).

To examine the influence of pulse width on tactile sensation, the median voltage amplitude for each subject was applied while gradually increasing the pulse width from 1 ms. Because the median amplitude lay in a transition region between tapping and pricking sensations, the perceived sensation type varied across subjects. Consequently, the perceptible pulse width range showed a relatively large standard deviation of 4.2 ms and a mean maximum pulse width of 6.93 ms (Figure S12a). Unlike voltage amplitude, which primarily determines the sensation type, pulse width modulates the depth and persistence of the perceived stimulus. Specifically, at a fixed median amplitude, increasing the pulse width enhanced both the magnitude and duration of the sensation. The recovery time of the current trace to baseline also increased with pulse width, extending the sustained perception of the stimulus. As shown in Figure S12b, the baseline recovery times were 0.3, 0.51, and 0.7 s for pulse widths of 1, 5, and 10 ms, respectively. Subjects reported that longer pulse widths were perceived as gentle, prolonged pressing. Interestingly, some subjects also described an apparently higher frequency, despite the actual stimulation frequency being fixed at 1 Hz. However, when the pulse width exceeded the critical duration, the sensation abruptly shifted to sharp pricking, characteristic of nociceptive activation.

The measured currents with varying pulse widths are shown in Figure S13a and analyzed in Figure S13b. Compared with amplitude modulation, pulse‐width modulation produced greater variability, with higher standard deviations in the measured currents. While current amplitudes for amplitude‐controlled stimulation ranged from 0 to 7.5 µA, pulse‐width modulation extended the range up to 20 µA, with a sharp increase near the pain threshold. These findings suggest that amplitude is better suited for finely adjusting perceived pressure magnitude and the sensation type, while pulse width controls the temporal persistence and intensity of a given sensation. Considering that the sensory threshold of the human forearm has been reported as 2–4 mN using von Frey hairs with a 0.5 mm tip diameter, corresponding to 10–20 kPa [53], and that the pressure‐pain threshold is approximately 250 kPa [54], the applied pain‐threshold voltage of 115.8 V in this study can be correlated with a pressure of 250 kPa.

### 2.6 Application

To enable practical use, system miniaturization should be considered. The function generator and voltage amplifier employed in Section 2.5 Electrotactile Stimulation are bulky and unsuitable for wearable, human‐interfacing applications. These components can be replaced by a microcontroller (Arduino Nano) and a high‐voltage stimulator (DRV2700), respectively. The Arduino Nano's output voltage is amplified by the DRV2700. Pulse width is controlled in firmware, and a 1 µF capacitor with a 1 kΩ resistor provides a low‐band‐pass filter to smooth the PWM‐generated voltage. Furthermore, the constant‐current supply for the Peltier element can be replaced by an Arduino Nano driving an H‐bridge current driver (BTS7960) powered by a three‐cell series battery pack (total 10.75 V). The Arduino Nano commands the BTS7960 to set polarity and apply current to the Peltier element. Given the maximum applied voltage of 10.75 V, current and polarity are fully controlled in firmware.

As shown in Figure 5a, the system consists of a sensing part and a stimulating part. The voltage signal from the multifunctional tactile sensor is conditioned by a differential amplifier (MCP602) and then processed by the Arduino Nano. The Arduino Nano estimates the applied pressure and temperature, and the extracted features are transmitted to the stimulation module, which consists of a controller and the actuators. For electrotactile output, the Arduino Nano synthesizes a pressure‐proportional waveform, and the DRV2700 amplifies it by 25 fold to match the voltage levels applied in Section 2.5 Electrotactile Stimulation. Then, the amplified pulse‐wave high voltage is applied to the electrotactile stimulator. In the same way, the BTS7960 applies the voltage with the appropriate polarity to the thermotactile stimulator based on temperature estimates computed by the Arduino Nano.

**FIGURE 5:**
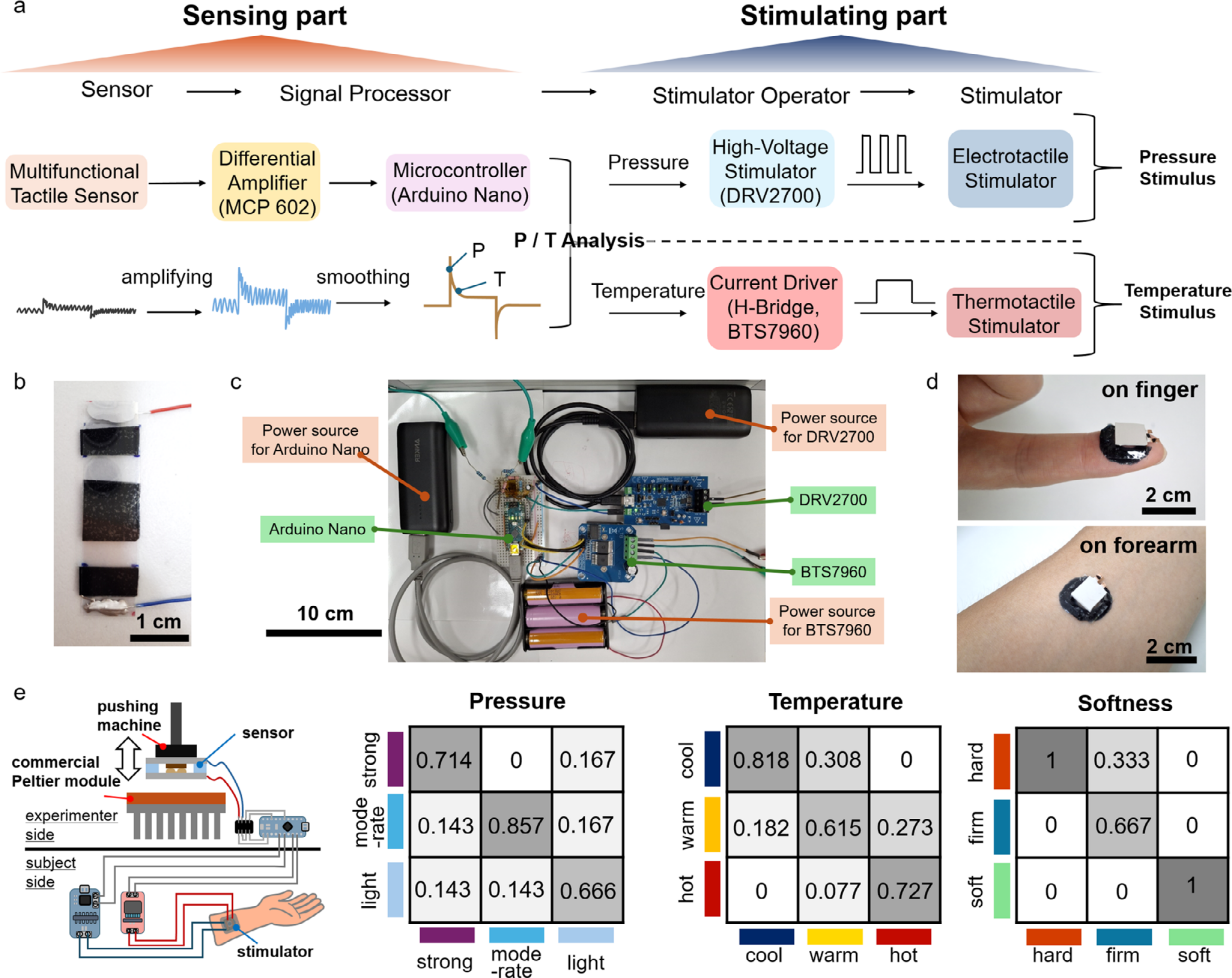
Electrical and psychophysical characterization of the electrotactile stimulator. (a) Schematic outline of the integrated system, illustrating the process from sensing applied pressure and temperature to delivering corresponding electrical stimulation through wearable stimulators. (b‐d) Hardware implementation of the integrated system, comprising the tactile sensor, signal processor, wearable stimulator, and power sources. (e) Accuracy of the system in discriminating pressure, temperature, and softness stimuli.

The tactile sensor is supported by a polyethylene terephthalate (PET) substrate, which can be mounted on various machines, and the wearable stimulator can be placed on the fingertip or forearm to deliver tactile sensations (Figure 5b,d). As shown in Figure 5c, power is applied by three separate sources for a portable system: a portable charger (Anker321, Anker Innovations Co., Ltd) powers the Arduino Nano and the DRV2700 individually, while a battery pack of three Li cells in series (Megacanon 3000, Shina C&C, Korea) powers the BTS7960 that sets the drive voltage and polarity for the thermotactile stimulator.

To evaluate system reliability, subjective psychophysical tests were conducted where the subject identified object properties based solely on tactile stimuli, without any visual cues. The tactile sensor was mounted on a pushing machine positioned above a commercial Peltier module, allowing for the controlled application of pressure and temperature. For each trial, the subject was tasked with identifying the magnitude of the stimulus perceived from the stimulators.

For the pressure discrimination task, the pushing machine applied three distinct pressures (light: 10 kPa, moderate: 50 kPa, strong: 90 kPa) to the sensor. The electrotactile stimulator's output voltage was linearly mapped to these pressures, ranging from a lower limit of 60 V to an upper limit of 80 V, consistent with previously established sensory and pain thresholds. After a brief familiarization phase where each condition was presented twice, each of the three pressure levels was presented twice, each of the three pressure levels was presented seven times in a randomized order.

For the temperature discrimination task, the Peltier module delivered cool, warm, and hot stimuli, which were generated by applying −1.0 V, +1.0 V, and +2.0 V to the thermotactile stimulator, respectively. An Arduino Nano processed the sensor's voltage signal, classifying temperature based on the decay slope: steeper negative slopes indicated a cooler stimulus, while shallower negative slopes indicated a hotter one. This classified temperature directly controlled the thermal stimulus delivered to the subject. Following a familiarization period, the cool and hot conditions were each presented eleven times, and the warm condition was presented thirteen times, all in a random sequence.

For the softness discrimination task, the pushing machine applied a constant 2.5 mm displacement to three objects of different compliance: hard glass, a hard sponge, and a soft sponge (Figure S14). As we previously reported [32], softer objects exert a lower opposing force under constant displacement, allowing for softness classification based on the measured pressure response. This pressure signal was then linearly mapped to an electrotactile stimulus, with the output voltage ranging from 60 to 80 V. After familiarization, each object was presented nine times in randomized order.

As shown in Figure 5e, for pressure discrimination, the correct identification rates for strong, moderate, and light pressure stimuli were 71.4%, 85.7%, and 66.6%, respectively. For temperature discrimination, the correct identification rates for cool, warm, and hot stimuli were 81.8%, 61.5%, and 72.7%, respectively. Misclassifications may stem from gradual desensitization under repeated electrotactile stimulation and from residual heat accumulation/thermal lag in the thermotactile stimulator, which can bias temperature judgements. For softness discrimination, subjects achieved accuracy of 100% for hard glass, 66.7% for hard sponge, and 100% for soft sponge, confirming the system's capability to distinguish objects of varying compliance.

Relative to prior psychophysical evaluations of the electrotactile stimulation, reported accuracies include 65% for intensity discrimination [55], 86% for distinguishing frequency levels with single‐pad feedback [56], 78.05% for classifying sensation quality (tapping, pricking, pressing, caressing) [57], and 93% perceptual accuracy under a fixed 100 kPa pressure [52]. Our mean accuracy of 81.7% for discriminating pressure level and softness is therefore comparable and lies within the published range. However, direct comparison is not strictly appropriate owing to methodological differences. For example, the study reported 86% varied frequency as the control parameter, whereas our study modulated amplitude. Likewise, the 93% result employed a 10 × 10 electrotactile array that was not affixed to the skin; when contact pressure for consistent contact was not applied, the accuracy of 93% dropped to 50%, highlighting sensitivity to contact conditions.

Importantly, our study also integrates a thermal channel, advancing beyond conventional pressure‐centric approaches limited to normal force or vibration [58]. By coupling thermal and pressure feedback, which is the key innovation, it delivers thermo‐mechanotactile cues from a single contact. This dual‐channel approach conveys richer perceptual information, enabling users to discern an object's thermal state and to make fine material discriminations—cues that are inaccessible through force alone.

To directly compare our integrated system with existing literature, we evaluated its psychophysical recognition accuracies against recent state‐of‐the‐art tactile stimulus‐replicating systems (Figure S15). For a fair comparison, we focused on studies that utilize electrotactile stimulation to evoke pressure‐like sensations, which is consistent with our methodology. Furthermore, we examined whether these existing platforms integrated tactile sensors to establish a fully closed‐loop pathway. Our analysis reveals that the recognition accuracies of our system are highly competitive, even when compared to literature dealing with separate electrotactile or thermotactile stimulations. Ultimately, this work successfully demonstrates a quantitatively evaluated, closed‐loop tactile stimulus‐replicating system that combines multimodal sensing with electro‐thermal stimulation.

## 3 Conclusion

We developed a stimulus‐replicating system that integrates a self‐powered multimodal tactile sensor with a wearable stimulator to reproduce mechanical and thermal cues on the skin. The tactile sensor, combining triboelectric and ionic mechanisms, simultaneously detects dynamic pressure, static pressure, and temperature without external power. The resulting multimodal signals directly drive the wearable stimulator, which integrates a flexible Peltier‐based thermotactile channel and a PEDOT:PSS/PU electrotactile electrode whose geometry was optimized via Multiphysics simulations for selective receptor activation. This integration enables faithful reproduction of tactile–thermal signatures that users perceive as realistic force–temperature sensations.

The electrotactile electrode maintained stable resistance across varying thermal conditions and selectively activated superficial mechanoreceptors with an optimized concentric geometry. For thermotactile stimulation, the AlN/PDMS composite encapsulation ensured both efficient heat transfer and mechanical compliance for the Peltier element. Furthermore, the acrylate‐based adhesive layer provided robust integration of the thermal and electrical stimulators. Psychophysical evaluation confirmed the system's reliability, demonstrating discrimination of pressure, temperature, and softness with an average accuracy of 78.5% (electrotactile: 81.7%, thermotactile: 72.0%), with misclassifications primarily attributed to sensory adaptation under repeated stimulation.

Despite the successful demonstration of the integrated system, certain limitations remain to be addressed for broader practical deployment: First, although the required voltage amplitude for electrotactile stimulation varies significantly across subjects, real‐time personalized voltage calibration was not implemented in the current setup. Future iterations must incorporate automated calibration algorithms to ensure consistent sensory experiences. Second, because the current sensing module is designed to detect absolute temperature, it is currently unable to replicate the transient heat flux associated with touching materials of different thermal conductivities (e.g., distinguishing between glass and metal at the same temperature).

Beyond a pressure‐centric haptic system, our study delivers bimodal feedback for more natural, context‐appropriate touch rendering. Receptor‐selective electrotactile stimulation enhances realism, and the flexible AlN/PDMS encapsulating layer of the thermotactile stimulator preserves wearability while achieving perceptible bidirectional (heating/cooling) actuation. Together with a compact design, the system maintains portability and user comfort without sacrificing stimulus richness.

Importantly, the shared sensing–stimulating architecture can establish a closed‐loop pathway in which real physical interactions are detected and faithfully conveyed to the user, providing a foundation for next‐generation bio‐mechatronic technologies. This biomimetic approach advances beyond predefined, virtual haptics toward realistic, context‐specific tactile reproduction. The proposed system not only enables nuanced perception in smart prosthetics—restoring tactile and thermal feedback to amputees—but also enhances tele‐haptic communication and robotic surgery by supplementing visual feedback with compliance and temperature cues.

## 4 Experimental Seciont/Methods

### 4.1 Materials

PU (Hydromed D4) was purchased from Advan Source Biomaterials. PEDOT:PSS (high‐conductivity grade, 3.0–4.0% aqueous dispersion), SA (contains 200 ppm monomethyl ether hydroquinone as inhibitor, 97%), LA (technical grade, 90%), AlN (powder, 10 µm, ≥ 98%), 2,2‐dimethoxy‐2‐phenylacetophenone (DMPA, 99%), and trichloro(1H,1H,2H,2H‐perfluorooctyl)silane (97%) were obtained from Sigma Aldrich, USA. PDMS (Sylgard 184 elastomer kit) was obtained from Dow Corning. UDA oligomer (CN9021, Sartomer) was obtained from Arkema, France. Cu tape and carbon tape were purchased from TERAOKA SEISAKUSHO Co., Ltd., Japan. Ethanol was acquired from Duksan General Science, South Korea, and deionized water was sourced from Sungwoo Genetech Co., Ltd., South Korea. Al foil (16 µm thickness) was purchased from Samjin Aluminum, South Korea. Silver resin paste (Elcoat P‐100) was obtained from CANS, Japan.

### 4.2 Fabrication of the Wearable Stimulator

The electrotactile electrode was fabricated as discussed in previous research.32 Fabricated PEDOT:PSS/PU solution was drop‐cast onto the Si wafer using a patterned polyimide (PI) film as shown in Figure S3a by a nanosecond pulsed fiber laser with a 355 nm wavelength (ULPN‐355‐6‐1‐6‐M, IPG Photonics, USA). Drop casted solution was dried on the flat plate under constant relative humidity of 25% for 24 h. Patterned electrode was lined up with Ag paste and Cu tape.

The thermotactile stimulator was fabricated with a Peltier element and flexible encapsulating layer. Peltier element was fabricated by locating four Peltier pellets obtained from a commercial Peltier module (TEM1‐12706), and they were connected electrically upside down with Cu tape pasted with Ag paste. 5.067 g of AlN powders and 6 g of PDMS were stirred until it got uniform color in an environment of 40% relative humidity to fabricate a 20 vol.% AlN/PDMS composite. After the composite was vacuum‐treated to eliminate the pores, the AlN/PDMS composite was blade‐coated on the glass that was self‐assembled monolayer (SAM) treated with trichloro(1H,1H,2H,2H‐perfluorooctyl)silane with 0.5 cm of PET spacer. Coated AlN/PDMS composite was dried at 70°C for 12 h. 0.5 cm thick cured AlN/PDMS composite was placed on another SAM‐treated glass with 1 cm of glass spacer. Additional uncured AlN/PDMS composite was poured onto the 0.5 cm thick cured AlN/PDMS composite, and before curing, previously fabricated 2 × 2 electrically connected Peltier pellets were placed onto the composite with gentle pressure so that the uncured AlN/PDMS composite surrounds each pellet to affix the Peltier elements onto the flexible encapsulating layer.

The adhesive layer was fabricated by mixing SA, LA, UDA, and DMPA in a weight ratio of 8: 72: 20: 0.5. The mixture was sonicated for 10 min until a transparent solution was obtained. The solution was then injected between two glasses separated by 300 µm polyimide tape spacers to define the layer thickness. Curing was performed for 2.5 h using a UV hand lamp (VL‐6LC, Vilber Lourmat, France) at a wavelength of 254 nm. After curing, the layer was released by separating the glass substrates.

### 4.3 Measurements

Material characterization was performed using a field emission scanning electron microscope (JEOL‐FE‐SEM, IT‐500HR). Electrochemical measurements were performed using a potentiostat (SP‐300, Biologic) and a digital multimeter (DMM 6500, Tektronix). Thermal characterization was conducted with a differential scanning calorimeter (Discovery DSC 250 Auto, TA Instruments). Material characterization was carried out using a Fourier‐transform infrared (FTIR) spectrometer (Invenio, Bruker). Mechanical properties were evaluated with a universal testing machine (UTM, WithLab, WL2100C) equipped with a 1 kgf load cell, as well as a custom bending apparatus. Pressure was uniformly applied to the sensor using a custom‐built pushing machine (Limotion Systems), while temperature stimulus was applied via a DC voltage supply (UP36‐6, TDK‐Lambda). Electrotactile stimulation was delivered using an arbitrary function generator (AFG1062, Tektronix) in combination with a high‐voltage linear amplifier (F20AD, Pendulum Instruments, Inc.)

### 4.4 Experiments With Human Subjects

Human subject experiments were conducted in compliance with all relevant ethical regulations under a protocol approved by the Yonsei University Institutional Review Board (application no. 7001988‐202508‐HR‐2864‐02). The study involved no additional risks to participants and adhered to the guidelines provided. A total of 15 healthy human subjects (9 male, 6 female, aged 21–35 years) provided written informed consent prior to participation. Before data collection, participants were trained with the assistance of experimenters to familiarize themselves with the electrotactile system and the sensation of electrical stimulation.

## Author Contributions

**Kyuhyun Hwang**: data curation, resources. **Jongbaeg Kim**: Writing – review and editing, supervision. **Ey‐In Lee**: conceptualization, investigation, validation, visualization, Writing – original draft. **Jin‐Woo Park**: conceptualization, validation, supervision, visualization, Writing – review and editing. **Chae‐Young Kang**: data curation, formal analysis.

## Funding

This work was supported by the National Research Foundation of Korea (NRF) grant funded by the Korean government (MSIT) (RS‐2025‐16064707).

## Conflicts of Interest

The authors declare no conflicts of interest.

## Supporting information




**Supporting File**: advs76616‐sup‐0001‐SuppMat1.docx.

## Data Availability

The data that support the findings of this study are available in the supplementary material of this article.
