# Supplementary material for: Wearable Electro‐Thermal Haptic Stimulator Driven by a Self‐Powered Tactile Sensor for Realistic Stimulus Replication
Source: Adv Sci (Weinh). 2026 Jul 13;13(56):e76616. doi: 10.1002/advs.76616 (PMC13360109; doi:10.1002/advs.76616)
Supplement: Supplementary file 1 [file ADVS-13-e76616-s001.docx]

Supporting Information

Wearable Electro-Thermal Haptic Stimulator Driven by a Self-Powered Tactile Sensor for Realistic Stimulus Replication

Ey-In Lee^1^, Chae-Young Kang^1^, Kyuhyun Hwang^2^, Jongbaeg Kim^2^, Jin-Woo Park^1^*

^1^Department of Materials Science and Engineering, Yonsei University, Seoul 03722, Republic of Korea

^2^School of Mechanical Engineering, Yonsei University, Seoul 03722, Republic of Korea

E-mail: [jwpark09@yonsei.ac.kr](mailto:jwpark09@yonsei.ac.kr) (J-W.P)

**Supplementary lists**

**Supporting Figure S1-15**

**Figure S1.** a) Simulation model of the concentric electrode positioned above the skin. b) Simulated electric field distribution in the skin under applied current.

 **Figure S2.** Activating function calculated through simulation for various concentric electrode designs with different inner electrode and outer electrode sizes

 **Figure S3.** a) Mold for fabricating outer electrode. b) Activating function obtained from simulation of the concentric electrode design with a 2 mm of inner electrode diameter and an 8 mm of outer electrode width.

 **Figure S4.** a) Stress-strain curves of AlN/PDMS composites fabricated under ambient (40% RH) and glove-box (moisture-free) conditions, compared with neat PDMS. b) FTIR spectra of AlN/PDMS composites with varying AlN loadings. c) FTIR spectra highlighting the absorption bands of AlN/PDMS composites fabricated under ambient (40% RH) and glove-box (moisture-free) conditions.

**Figure S5.** Cyclic bending test of a thermotactile stimulator at a 20 mm radius.

**Figure S6.** a) Cross-sectional SEM images (top) of the layer before and after the thermal cycling test. The corresponding EDS mapping images (bottom) of the layer after the thermal cycling test. b) Transient temperature profiles of the layer under the two conditions (before and after the thermal cycling test) during the thermal application from 20 ℃ to 37 ℃.

 **Figure S7.** a) SA/LA mixture with 70wt% SA before UV curing. b) Adhesion properties of SA/LA adhesive (200 μm thickness).

**Figure S8.** EIS data from human subjects.

 **Figure S9.** a) Pulse-wave voltages applied to the subject with varying amplitudes and pulse widths. b) Example of pulse-wave voltage generated by function generator. c) Equivalent circuit model emulating voltage application to the skin. d) Current response measured by applying pulse-wave voltage to the equivalent circuit.

 **Figure S10.** Table summarizing the sensory and pain thresholds of individual subjects.

 **Figure S11.** Current responses measured from individual subjects under voltage stimulation with varying amplitudes.

 **Figure S12.** a) Table summarizing the pulse-width pain thresholds of individual subjects. b) Current responses measured from individual subjects under voltage stimulation with varying pulse widths.

**Figure S13.** a) Current responses measured from individual subjects under voltage stimulation with varying pulse widths. b) Current response under different applied voltage pulse widths (N=11).

**Figure S14.** Hard glass, hard sponge, soft sponge for softness discriminating evaluation.

 **Figure S15.** Comparison of tactile tactile stimulus-replicating systems based on electrotactile and thermotactile stimulations.
